# Atmospheric non-thermal plasma inactivation of *Ascosphaera apis*, the causative agent of chalkbrood disease in honeybee

**DOI:** 10.1038/s41598-024-52221-1

**Published:** 2024-01-21

**Authors:** Thummanoon Boonmee, Chainarong Sinpoo, Kunlada Thayatham, Pradoong Suanpoot, Terd Disayathanoowat, Jeffery S. Pettis, Veeranan Chaimanee

**Affiliations:** 1 Department of Agro-Industrial Biotechnology, Maejo University Phrae Campus, Phrae, 54140 Thailand; 2 Bee Protection Laboratory, Department of Biology, Faculty of Science, Chiang Mai University, Chiang Mai, 50200 Thailand; 3 Office of Research Administration, Chiang Mai University, Chiang Mai, 50200 Thailand; 4 Research Center of Deep Technology in Beekeeping and Bee Products for Sustainable Development Goals (SMART BEE SDGs), Chiang Mai University, Chiang Mai, Thailand; 5 Department of Forest Industry Technology, Maejo University Phrae Campus, Phrae, 54140 Thailand; 6 Pettis and Associates LLC, Salisbury, MD 21801 USA

**Keywords:** Biotechnology, Microbiology

## Abstract

*Ascosphaera apis* is a worldwide pathogenic fungi of honeybees that can cause a decline in bee populations. In this study, we investigated the antifungal activity of non-thermal plasma on fungal growth. Spore inactivation after exposure to gas plasma by liquid phase and plasma activated water (PAW) and pathogenicity of *A. apis *in vivo were also examined. The results demonstrated that the mycelial growth of fungi was completely inhibited after argon plasma treatment. Both gas plasma and PAW exposures resulted in a significant decrease of *A. apis* spore numbers, maximum reduction of 1.71 and 3.18-fold, respectively. Germinated fungal spores on potato dextrose agar were also reduced after plasma treatment. SEM analysis revealed a disruption in the morphological structure of the fungal spores. The pathogenicity of *A. apis* on honeybee larvae was decreased after spores treated by gas plasma and PAW with a disease inhibition of 63.61 ± 7.28% and 58.27 ± 5.87%, respectively after 7 days of cultivation. Chalkbrood in honey bees have limited control options and our findings are encouraging. Here, we demonstrate a possible alternative control method using non-thermal plasma for chalkbrood disease in honeybees.

## Introduction

Chalkbrood disease is a fungal disorder in honeybee brood, caused by *Ascosphaera apis*^1,2^. This disease has spread worldwide and increased in recent years^3^. *A. apis* can infect brood of any caste and larvae, 1–4 day-old, are more susceptible^4–6^. Transmission of the disease can occur within the colony via food sharing and between colonies during beekeeper management^7^. Ingested spores germinate in the lumen and start penetrating the peritrophic membrane of the midgut^8^. The fungal mycelium grows inside of the body and breaks out through the posterior end of the larva^9^. Dead honeybee larvae can be either white or black mummies, depending on ascospore production^10^. Chalkbrood causes a loss in bee population and a loss in colony productivity have been reported^4,11^. Currently, control of chalkbrood disease is by using chemicals and natural compounds, improving genetic stock and management and sanitation strategies^10,12–16^. Although these control strategies seem to be effective against chalkbrood disease, some drawbacks such as chemical residues in honey and bee hives, chemical resistance, and impact on honeybees are still of concern^17^. New technologies and/or alternative strategies for chalkbrood disease control are needed in beekeeping.

Atmospheric non-thermal plasma technology has gained attention as a potential tool for decontamination of microorganisms. It is widely used in medicine, food, and agriculture^18–21^. Plasma generates reactive oxygen and nitrogen species (RONS) (such as OH, O, O_3_, H_2_O_2_, NO, NO_2_^−^ and NO_3_^−^), UV, ions and charged particles^18,22^. Gas plasma and plasma activated water (PAW) on microbial inactivation has been reported in *Fusarium fujikuroi*, SARS-CoV-2, *Aspergillus brasiliensis*, *Staphylococcus aureus*, *Escherichia coli*, *Colletotrichum asianum*^20,23–28^. The reactive species generated by plasma depend on discharge type, working gas and environmental conditions that are responsible for microbial inhibition^24,26^. However, the efficacy of non-thermal plasma on honeybee pathogens has not been evaluated.

In this study, the fungicidal activity of atmospheric non-thermal plasma on chalkbrood disease agent, *A. apis* in honeybees was investigated. Both gas plasma and PAW was performed for their effects on fungal mycelium and spores. Physicochemical properties of PAW were carried out to determine how PAW can contribute to the fungal inactivation. The pathogenesis of fungal spores after plasma treatment was also investigated in vivo. Our results may provide useful information on plasma application for disease control in honeybees.

## Results

### Optical emission spectra

**Figure 1:**
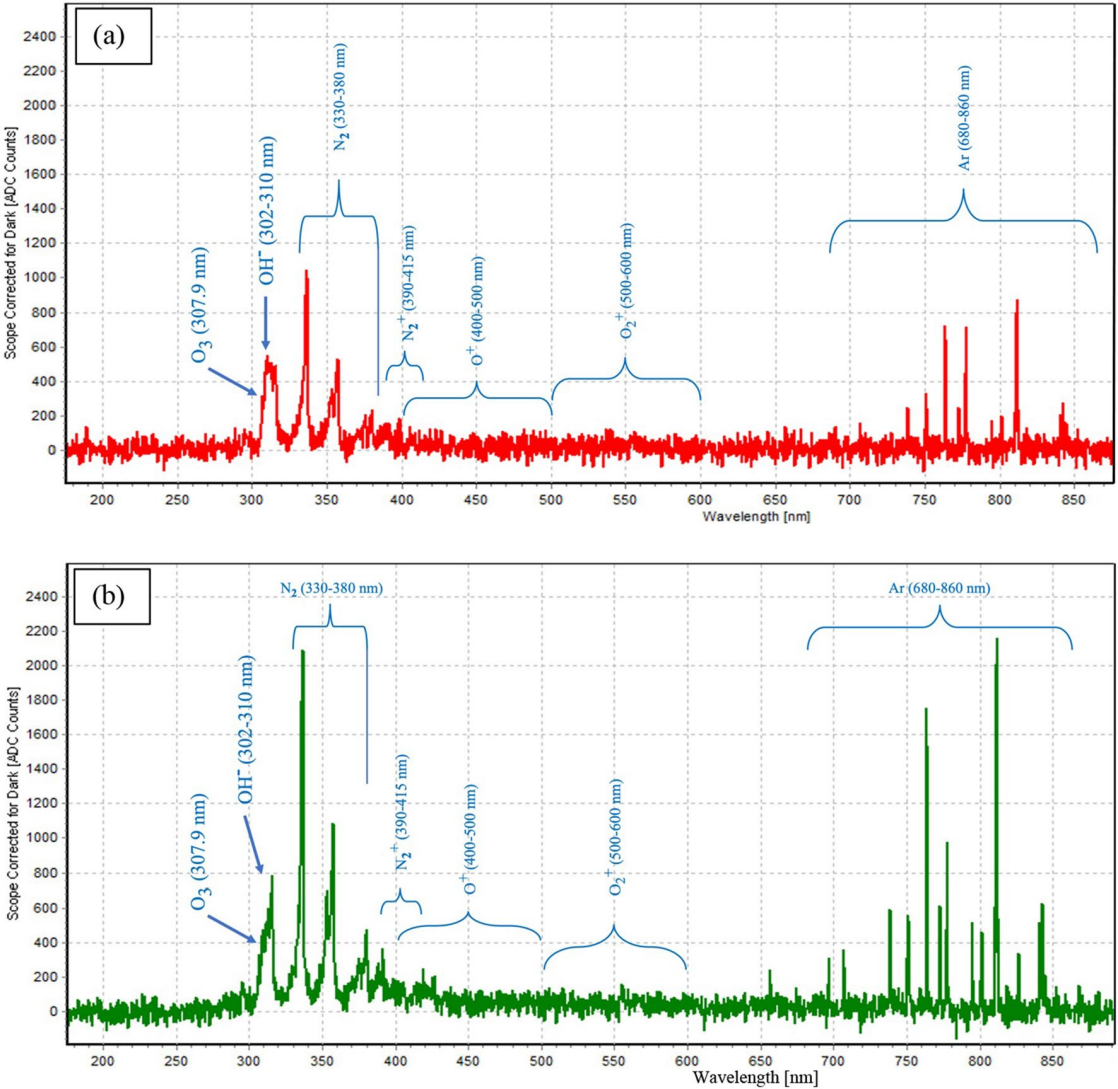
Typical optical emission spectrum (OES) of argon plasma jet at gas flow rate of 0.25 Lpm (**a**) and 0.5 Lpm (**b**).

The emission spectrum is presented in Fig. 1. The excited species, O_3_, OH^–^, N_2_, N_2_^+^, O^+^, O_2_^+^ and Ar were detected at 307.9 nm, 302–310 nm, 330–380 nm, 390–415 nm, 400–500 nm, 500–600 nm and 680–860 nm, respectively. OES results are dominated by the N_2_ second positive system at 330–380 nm. The high intensity of N_2_ was obtained by increasing the argon flow rate.

### Determination of physicochemical properties of PAW

Physicochemical parameters of PAW are shown in Table 1. Physicochemical properties were different between plasma treatments. The PAW’s pH decreased to 5.99–6.67 from 7.16. The pH was decreased with the increasing treatment time. The concentrations of H_2_O_2_ in freshly prepared PAW were dramatically increased. PAW generated by argon at flow rate of 0.25 Lpm for 3, 5 and 10 min had H_2_O_2_ concentration of 80, 100 and 200 mg/L, respectively. Higher concentrations of H_2_O_2_ (100–300 mg/L) were obtained by increasing the gas flow rate to 0.50 Lpm. However, NO_2_^−^ and NO_3_^−^ was not detected in water after plasma treatment under set conditions. Conductivity was decreased after the argan plasma treatment.

**Table 1:** Physicochemical properties of PAW generated by argon gas at different flow rate and time duration (n = 3/treatment).

Treatment (gas flow rate)	Treatment time (min)	pH	H_2_O_2_ (mg/L)	Nitrite (mg/L)	Nitrate (mg/L)	Conductivity (μS/cm)	mV (electrode)
Control (untreated)	–	7.16 ± 0.04	0.00 ± 0.00	Not detected		27.07 ± 1.10	−2.33 ± 0.88
0.25 Lpm	3	6.67 ± 0.01	80.00 ± 0.00	Not detected		14.27 ± 0.70	35.00 ± 2.52
	5	6.45 ± 0.01	100.00 ± 0.00	Not detected		22.50 ± 0.85	43.00 ± 1.15
	10	6.15 ± 0.03	200.00 ± 0.00	Not detected		18.67 ± 0.73	48.33 ± 1.20
0.50 Lpm	3	6.65 ± 0.05	100.00 ± 0.00	Not detected		9.10 ± 0.70	27.67 ± 1.45
	5	6.63 ± 0.05	200.00 ± 0.00	Not detected		9.50 ± 0.46	32.00 ± 0.58
	10	5.99 ± 0.02	300.00 ± 0.00	Not detected		13.63 ± 1.09	68.33 ± 3.28

### Fungicidal efficiency of argon plasma on *A. apis* mycelial growth

**Figure 2:**
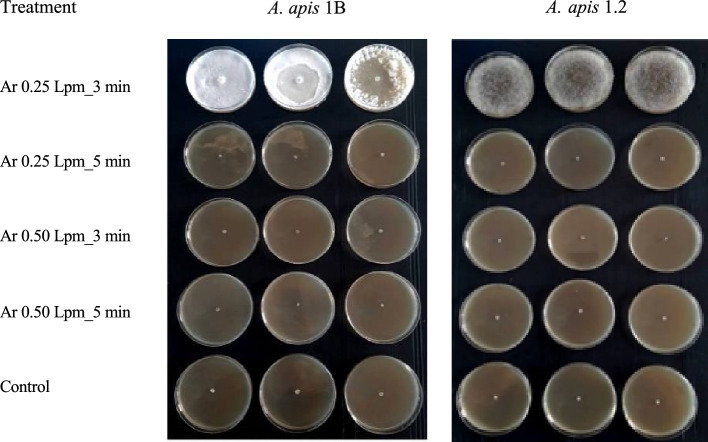
Mycelial growth of *A. apis* 1B and *A. apis* 1.2 at day 8 after argon plasma treatment at different gas flow rate (0.25 and 0.50 Lpm) and treatment duration (3 and 5 min).

The mycelial growth of *A. apis* 1B and *A. apis* 1.2 was totally inhibited for 8 days after plasma treatments at argon flow rate of 0.25 and 0.50 Lpm for 3 and 5 min (Table 2 and Fig. 2). The mycelia of control untreated fungal strains grew continually until they reached the edge of the petri dish.

**Table 2:** The mycelial growth of *A. apis* 1B and *A. apis* 1.2 after argon plasma treatment for 8 days of incubation (n = 3/treatment).

Fungal strain	Treatment	Mycelium growth* (mm)							
		Day 1	Day 2	Day 3	Day 4	Day 5	Day 6	Day 7	Day 8
*A. apis* 1B	Ar 0.25 Lpm/3 min	0.00 ± 0.00^a^	0.00 ± 0.00^a^	0.00 ± 0.00^a^	0.00 ± 0.00^a^	0.00 ± 0.00^a^	0.00 ± 0.00^a^	0.00 ± 0.00^a^	0.00 ± 0.00^a^
	Ar 0.25 Lpm/5 min	0.00 ± 0.00^a^	0.00 ± 0.00^a^	0.00 ± 0.00^a^	0.00 ± 0.00^a^	0.00 ± 0.00^a^	0.00 ± 0.00^a^	0.00 ± 0.00^a^	0.00 ± 0.00^a^
	Ar 0.50 Lpm/3 min	0.00 ± 0.00^a^	0.00 ± 0.00^a^	0.00 ± 0.00^a^	0.00 ± 0.00^a^	0.00 ± 0.00^a^	0.00 ± 0.00^a^	0.00 ± 0.00^a^	0.00 ± 0.00^a^
	Ar 0.50 Lpm/5 min	0.00 ± 0.00^a^	0.00 ± 0.00^a^	0.00 ± 0.00^a^	0.00 ± 0.00^a^	0.00 ± 0.00^a^	0.00 ± 0.00^a^	0.00 ± 0.00^a^	0.00 ± 0.00^a^
	Control	57.73 ± 13.66^b^	64.07 ± 10.47^b^	69.58 ± 7.78^b^	73.85 ± 5.68^b^	78.28 ± 3.50^b^	85.00 ± 0.00^b^	85.00 ± 0.00^b^	85.00 ± 0.00^b^
*A. apis* 1.2	Ar 0.25 Lpm/3 min	0.00 ± 0.00^a^	0.00 ± 0.00^a^	0.00 ± 0.00^a^	0.00 ± 0.00^a^	0.00 ± 0.00^a^	0.00 ± 0.00^a^	0.00 ± 0.00^a^	0.00 ± 0.00^a^
	Ar 0.25 Lpm/5 min	0.00 ± 0.00^a^	0.00 ± 0.00^a^	0.00 ± 0.00^a^	0.00 ± 0.00^a^	0.00 ± 0.00^a^	0.00 ± 0.00^a^	0.00 ± 0.00^a^	0.00 ± 0.00^a^
	Ar 0.50 Lpm/3 min	0.00 ± 0.00^a^	0.00 ± 0.00^a^	0.00 ± 0.00^a^	0.00 ± 0.00^a^	0.00 ± 0.00^a^	0.00 ± 0.00^a^	0.00 ± 0.00^a^	0.00 ± 0.00^a^
	Ar 0.50 Lpm/5 min	0.00 ± 0.00^a^	0.00 ± 0.00^a^	0.00 ± 0.00^a^	0.00 ± 0.00^a^	0.00 ± 0.00^a^	0.00 ± 0.00^a^	0.00 ± 0.00^a^	0.00 ± 0.00^a^
	Control	35.65 ± 0.30^b^	53.47 ± 0.68^b^	69.45 ± 0.76^b^	79.47 ± 0.69^b^	85.00 ± 0.00^b^	85.00 ± 0.00^b^	85.00 ± 0.00^b^	85.00 ± 0.00^b^

*Different letters indicate significant difference among treatment groups (Kruskal–Wallis test, Steel–Dwass posthoc; p < 0.05).

### Argon plasma inactivates *A. apis* spores

**Figure 3:**
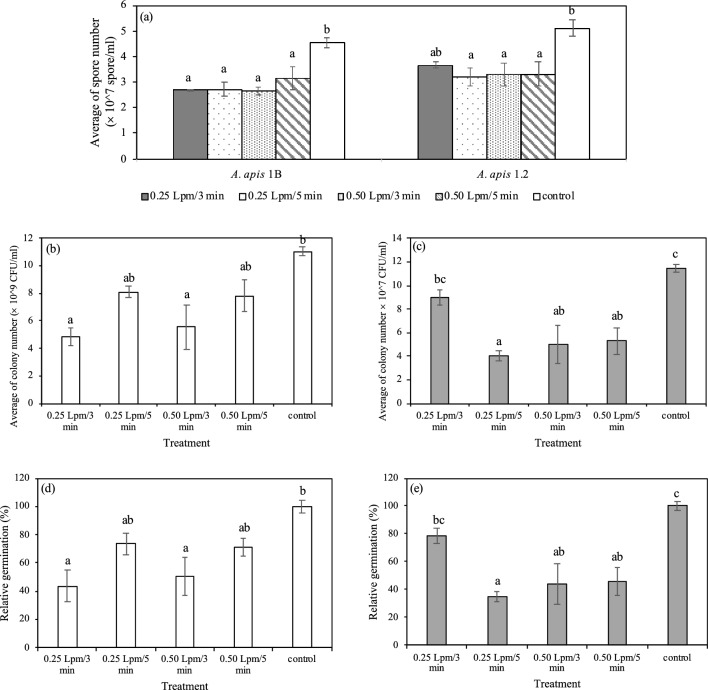
*A. apis* spore count using hemocytometer (**a**), number of germinated spores of *A. apis* 1B (**b**) and *A. apis* 1.2 (**c**) on PDA agar after argon plasma treatment by liquid phase and relative spore germination percentage of *A. apis* 1B (**d**) and *A. apis* 1.2 (**e**) following plasma treatment compared to that of control (untreated). Different letters indicate significant differences between treatment groups (ANOVA, Tukey-HSD; p < 0.05).

The spore and colony count were applied to investigate the fungicidal activity of plasma on *A. apis* spores. Average of spore number of *A. apis* 1B was significantly decreased after plasma treatments (ANOVA, p = 0.002) (Fig. 3a). Spore numbers declined about 1.44–1.71 folds when compared to untreated control. However, colony count in the treatments of gas flow rate of 0.25 Lpm for 3 min and 0.50 Lpm for 3 min were significantly different when compared with untreated control (ANOVA, p = 0.0121) (Fig. 3b) with the relative germination of 43.67% and 50.33%, respectively (Fig. 3d). Plasma generated by argon at flow rate of 0.25 Lpm for 5 min and 0.50 Lpm for 3 and 5 min reduced the spore number of *A. apis* 1.2 (ANOVA, p = 0.0195) (Fig. 3a). Argon plasma showed the higher effects on *A. apis* 1.2 inactivation, as its CFU was reduced 2.16–2.85 times (Fig. 3c). Approximately 35–46% relative germination was observed in plasma-treated spores under these conditions (Fig. 3e).

### Efficiency of PAW in *A. apis* spore inactivation

**Figure 4:**
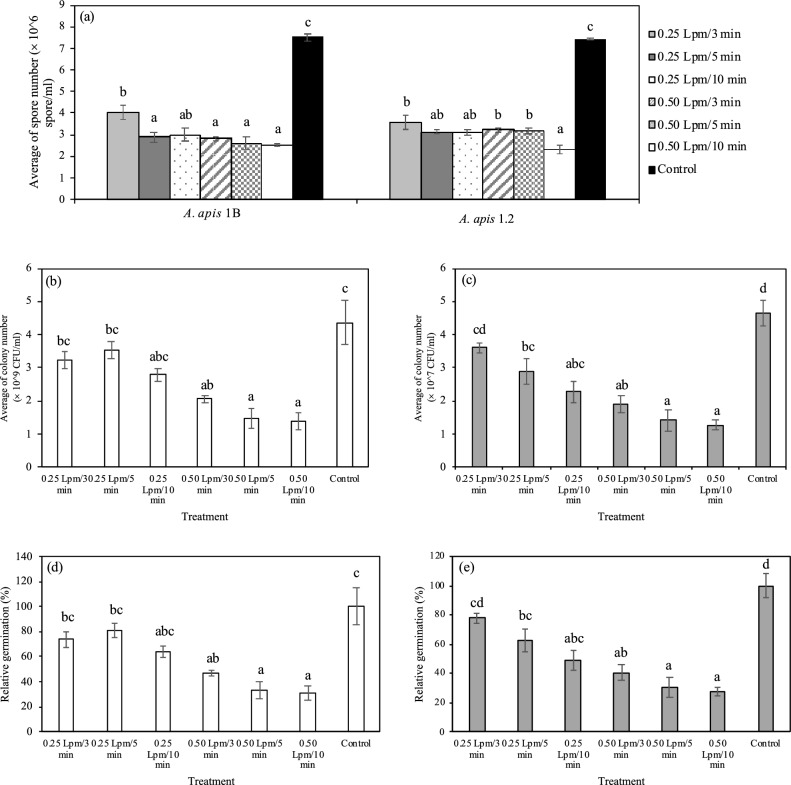
*A. apis* spore count using hemocytometer (**a**), number of germinated spores of *A. apis* 1B (**b**) and *A. apis* 1.2 (**c**) on PDA agar after plasma activated water (PAW) treatment and relative spore germination percentage of *A. apis* 1B (**d**) and *A. apis* 1.2 (**e**) following PAW treatment compared to that of control (untreated). Different letters indicate significant differences between treatment groups (ANOVA, Tukey-HSD; p < 0.05 and Kruskal–Wallis test, Steel–Dwass posthoc; p < 0.05).

PAW generated by argon had a strong fungicidal effect on both strains of *A. apis*. Spore numbers were reduced in all plasma treatments with approximately 1.85–2.98 and 2.09–3.18-fold reduction of *A. apis* 1B and *A. apis* 1.2, respectively (Fig. 4a). Fungicidal activity of PAW increased with increasing gas flow rate and exposure time. Colony count was assessed after 1 h of incubation, and fungal spore germination was significantly inhibited (approximately 31.33–33.67% and 27.33–30.33% relative germination for *A. apis* 1B and *A. apis* 1.2, respectively) by argon flow rate of 0.50 Lpm for 5 and 10 min (Fig. 4b–e).

### Morphology of *A. apis* spore

**Figure 5:**
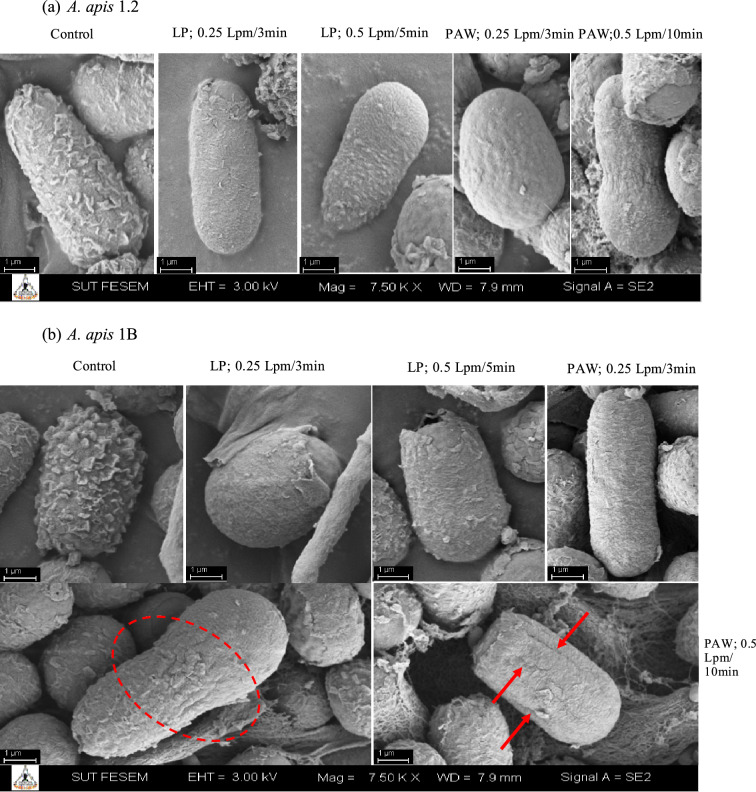
Scanning electron microscopy (SEM) images of *A. apis* spores; *A. apis* 1.2 (**a**) and *A. apis* 1B (**b**) after exposed to gas plasma by liquid phase (LP) and plasma activated water (PAW) at different gas flow rate and duration (magnification, ×7500).

SEM images of *A. apis* spore before and after argon plasma and PAW treatments are shown in Fig. 5. The untreated spores were intact, with a rough surface. After plasma treatment, spore morphology was ruptured, distorted, and changed to a smooth surface (Fig. 5). Both fungal spores shrank after being treated with PAW generated by argon flow rate of 0.5 Lpm for 10 min (Fig. 5a, b).

### Argon plasma reduces *A. apis* infection

**Figure 6:**
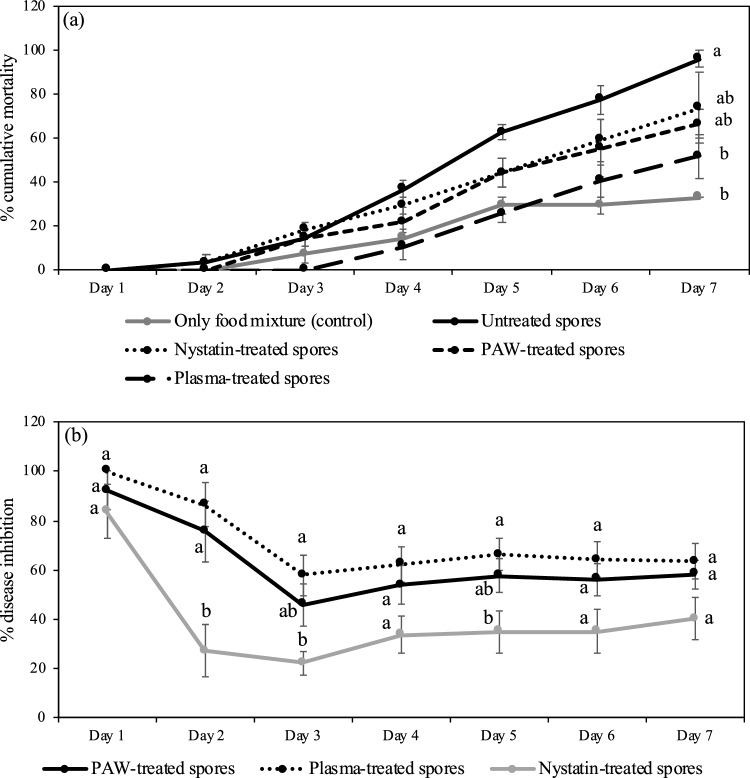
Percentage of cumulative (mean ± SE) honeybee larvae mortality (n = 27/treatment) (**a**) and percentage of chalkbrood disease inhibition (mean ± SE) (n = 9/treatment) after *A. apis* 1.2 spores treated with argon plasma by liquid phase and plasma activated water (PAW) and nystatin for 7 days. Different letters indicate statistically significant differences (ANOVA, Tukey-HSD; p < 0.05 and Kruskal–Wallis test, Steel–Dwass posthoc test; p < 0.05). Comparisons were made between treatment at each time interval.

Since fungal spores were inactivated by argon plasma and PAW, *A. apis* 1.2 infection in honeybee larvae might be decreased. Spores were treated with a gas flow rate of 0.25 Lpm for 5 min and PAW generated by gas flow rate of 0.50 Lpm for 10 min. Percentage of cumulative mortality of honeybee larvae in plasma and PAW treatments was lower than the untreated controls and spores treated with nystatin (Fig. 6a and Table in S1 Table). Fungal infection was evaluated by measuring mycelial growth on PDA from treated larvae after being fed with *A. apis* spores and food mixture for 7 days. The mycelial growth of spores treated with argon plasma and PAW showed significant differences when compared with untreated spores during the time periods (Table 3). Compared to nystatin-treated spores, mycelium of *A. apis* after plasma and PAW treatments grew slowly on PDA media on days 2–3 and it was not different on days 4–7 of experiment. Percent of disease inhibition was calculated. The results demonstrated that spores treated with argon plasma and PAW had a 63.61 ± 7.28% and 58.27 ± 5.87% disease inhibition on day 7 (Fig. 6). While the percent of inhibition was 40.09 ± 8.47% in nystatin-treated spores (Fig. 6b). However, there was no significant difference.

**Table 3:** The mycelial growth of *A. apis* 1.2 on PDA plate for 7 days (n = 9/treatment) of treated honeybee larvae after being inoculated with spores treated in liquid phase and PAW by argon plasma at gas flow rate of 0.25 Lpm for 5 min and 0.50 Lpm for 10 min, respectively.

Treatment	Mycelium growth* (mm)						
	Day 1	Day 2	Day 3	Day 4	Day 5	Day 6	Day 7
Royal jelly (control)	0.00 ± 0.00^a^	0.00 ± 0.00^a^	0.00 ± 0.00^a^	0.00 ± 0.00^a^	0.00 ± 0.00^a^	0.00 ± 0.00^a^	0.00 ± 0.00^a^
Spore suspension (untreated)	5.14 ± 0.26^b^	6.89 ± 0.46^b^	9.75 ± 0.84^d^	15.26 ± 0.73^d^	20.73 ± 0.91^d^	25.39 ± 0.77^d^	31.86 ± 1.67^c^
PAW-treated spores	0.41 ± 0.41^a^	1.68 ± 0.84^a^	5.28 ± 0.84^bc^	7.03 ± 1.21^bc^	8.77 ± 1.39^bc^	11.10 ± 1.68^bc^	13.29 ± 1.87^b^
Plasma-treated spores	0.00 ± 0.00^a^	0.93 ± 0.62^a^	4.09 ± 0.80^b^	5.72 ± 1.10^b^	6.99 ± 1.37^b^	9.08 ± 1.80^b^	11.59 ± 2.32^b^
Nystatin-treated spores	0.84 ± 0.56^a^	5.26 ± 0.82^b^	7.88 ± 0.68^ cd^	10.39 ± 1.35^c^	13.80 ± 1.97^c^	16.66 ± 2.38^c^	19.09 ± 2.70^b^

*Different letters indicate significant difference among treatment groups (Kruskal–Wallis test, Steel–Dwass posthoc; p < 0.05).

## Discussion

Atmospheric pressure plasma technology has gained attention in recent years for antimicrobial activity and its applications in many industries. Both direct plasma and PAW have been reported as potential disinfection agents, including fungal inactivation. Here, we first studied in vitro and in vivo the antifungal activity of argon plasma and PAW on the causative agent of chalkbrood disease, *A. apis,* in honeybees. Our results demonstrate that plasma and PAW generated by argon gas at different flow rates and time exposure can result in differing levels of fungal inactivation. The efficiency of antimicrobial activity depends on the chemical species generated by plasma which rely on gas feeding, flow rate, treatment time and environmental factors and microbial species^25,29,30^. In these studies, we demonstrate that the mycelial growth of *A. apis* can be totally inhibited by argon plasma. The hydroxyl molecules and ozone are produced with a high intensity. Although OH radicals probably do not play a key role in the inhibition process by itself, the combination of two OH molecules (hydrogen peroxide, H_2_O_2_) can cause microbicidal effects^31^. Thus, these reactive oxygen species generated by argon plasma could play important roles in fungal inactivation. Several studies have reported that ROS and RNS produced by plasma play crucial roles in microbial inactivation^23–25^. The reactive species can cause oxidative stress, lipid peroxidation, enzyme inactivation, cell leakage and DNA damage^32–34^.

Our results demonstrate that both direct plasma and PAW could reduce fungal spores. According to the concentrations of H_2_O_2_ were highly detected in water after plasma treatment, this chemical might be play an important role for fungal inactivation. The exposure to high concentration of H_2_O_2_ could result in spore burst. Moreover, direct plasma treatment against suspensions of *A. apis* spores was less effective than PAW under the same conditions. This might be due to the lower final concentration of *A. apis* (5 × 10^6^ spores/ml) used for PAW treatment. We suggest that it probably increases the interactions between the reactive species and fungal spores. Meanwhile, fungicidal inactivation of PAW was highly related to the treatment time since the amount of chemically reactive species (H_2_O_2_) increased with treatment time. In contrast, Los et al.^35^ reported that air plasma was more efficient against suspensions of *Aspergillus flavus* than PAW. In recent years, the potential of PAW as a fungicidal disinfectant has been studied^26,28,36,37^. Many factors could contribute to the different efficacies reported above. Kang et al.^23^ demonstrated that the efficiency of plasma depends on fungal spore forms. Arc discharge plasma inactivates *Fusarium Fujiko* spores infected rice seeds more efficiently than spores submerged in water. Radical species generated by plasma have lethal effects on fungal spores including disruption of cell wall leads to the release of intracellular components, lipid peroxidation and DNA damage^37^. In this study, there was a change in spore envelops in the direct plasma-treated spores and PAW-treated spores compared with that of control spores. The damage to spores increased with increasing plasma duration. Our data suggest that plasma inactivation of fungal spores maybe primarily due to spore envelop damage and subsequent leakage of intracellular components. Although antibacterial effects of plasma are stronger than fungicidal inactivation because of the differences in cell structure and components, the actions occurring with the cells are compatible^28,29,34^. The mechanism of plasma action is mainly based on the damage to the cell wall and cell membrane structures, leading to a leakage of the cytoplasmic components^35,37,38^. Moreover, plasma can cause DNA damage, protein oxidation, lipid peroxidation and cell apoptosis ^38–41^. Therefore, the mechanisms at subcellular level should be further investigated for a better understanding of the inactivation mechanism during plasma treatments.

In addition, our data showed that the ability of *A. apis* spores treated by plasma and PAW to infect honeybee brood was lower than nystatin-treated spores. This suggests that some spores, interrupted by plasma, have lost the ability for reproduction.

Although, several studies have been reported the efficacy of plasma on microbial inactivation, the current study reported for the first time the fungicidal activity against honeybee pathogen, *A. apis*. Overall, we clearly demonstrated that both argon plasma and PAW treatments can be effective tools against *A. apis* spores in honeybees. These treatments could significantly inactivate the fungal mycelium, spore viability and chalkbrood disease infection. These results demonstrate the efficacy of plasma in inactivation of fungal spores in the laboratory. However, several factors must be considered for beekeeping application. Honeybee colonies offer a very challenging environment in which to apply many control compounds but our results call for further study in the application of PAW or plasma. The environmental conditions such as humidity and temperature and the complexities of plasma and PAW require extensive investigation to understand the mechanisms of inactivation in different environmental states. Studies on the effects of plasma and PAW on honeybees and the application of these tools to control disease in beehives also needs to be further investigated. Both argon plasma and PAW treatments could be used in beekeeping to reduce the impact of chalkbrood on honeybee colonies especially PAW. Some ideas are their use in cleaning of used beeswax combs, sterilization of beeswax or other as yet undetermined applications in beekeeping. A challenge for developing gas plasma and PAW, especially PAW in the beekeeping is to optimize the factors of plasma generation to obtain highly efficient fungicidal activity. Moreover, stabilizers or additives should be considered to maintain the longevity of fungicidal chemicals without compromising their effectiveness. Also, the delivery system of PAW to beehives, the effect of plasma and PAW on bee health must be investigated. These innovations can lead to the develop of efficient and eco-friendly control strategies for honeybee diseases.

## Materials and methods

### Plasma device and plasma activated water (PAW) generation

**Figure 7:**
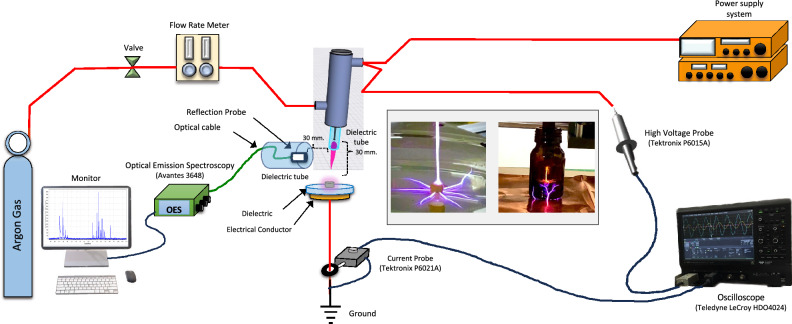
Schematic diagram of plasma set up.

**Figure 8:**
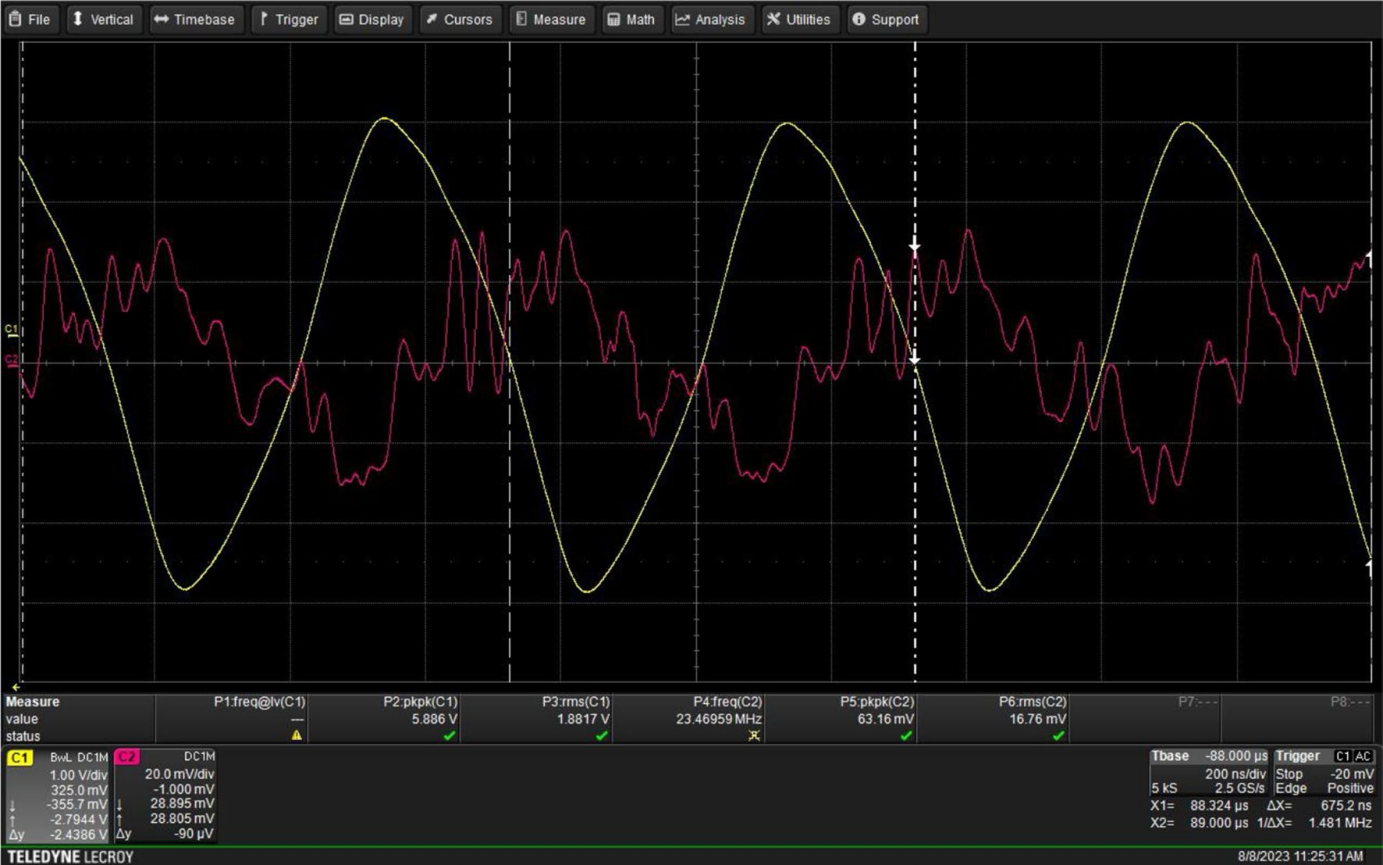
Typical waveforms of the discharge current and voltage.

A schematic diagram of the atmospheric-pressure plasma jet system used in this study is shown in Fig. 7. Plasma was generated by argon gas based on gliding arc discharge in air with a 5.886 kV input voltage and a 1.481 MHz frequency (Fig. 8). The different flow rates of gas were applied and monitored by a mass-flow controller. The electrical properties were measured by a high-voltage probe P6015A (Tektronix, USA). The voltage and current waveforms were recorded using an oscilloscope HDO4024 (Teledyne LeCroy, USA) with a 200 MHz bandwidth and a 2.5 GS/s sampling rate. The spectra of plasma jet emission were determined by Optical Emission Spectroscopy (AVANTES, USA).

PAW was produced by placing the 5 ml of sterile deionized water in the glass tube under the jet nozzle. The working distance between a nozzle and water surface was 20 mm. Argon gas with flow rate of 0.25 and 0.50 Litre per minute (Lpm) were applied with the exposure time of 3, 5 and 10 min.

### Fungal strains, culture condition and spore preparation

Two strains of *A. apis* were used in this study. Fungal strains were isolated from dry mummies collected in Beltsville, Maryland in 2015, identified by sequencing of internal transcribed spacer (ITS) and maintained at –20 °C in 20% (v/v) glycerol^16^. Each isolate was inoculated on potato dextrose agar (PDA) and incubated at room temperature for 7 days to obtain mycelia for plasma treatment. Spore suspensions were prepared by adding 10 ml of sterile water on the 10-day-old cultures grown on PDA and scraping spores off from mycelia with a sterile spreader. Then, suspension was adjusted with sterile water to 5 × 10^7^ spores/ml for experiments.

### Plasma treatment of *A. apis* mycelial growth

A mycelial plug (6 mm) was cut using a cork borer from a seven-day old of fungal mycelium and transferred to a sterile petri dish. Three mycelial plugs (replicates) were placed in a plate. Plates containing fungal plugs were placed under an atmospheric-pressure plasma jet nozzle with a working distance of 20 mm. The gas flow rate at 0.25 and 0.50 Lpm for 30 and 60 s were assigned as treatment. Untreated-plasma mycelia were used as control. After plasma treatment, mycelial plugs were transferred and individually placed on PDA plates using sterile forceps. PDA plates were incubated at room temperature and mycelial growth was measured daily until mycelia reached the edge of the plates^36^. Mycelial growth was measured along two diameters of colony at right angles to one another and the average diameter calculated^42^.

### Plasma treatment of *A. apis* spore

For determination of the antifungal effect on *A. apis* spores, 1 ml of spore suspensions with a concentration of 5 × 10^7^ spores/ml, in glass tubes, were directly exposed to plasma. The plasma jet nozzle was placed at a distance of 20 mm from the surface of suspension (Fig. 7). Argon gas flow rate at 0.25 and 0.50 Lpm for 3 and 5 min were treated. Untreated-plasma spores were assigned as control. Afterwards, the fungal spore suspension was incubated at 30 °C for 1 h. All treatments were repeated three times for statistical analysis. Spores were counted using a hemocytometer and spore suspensions were further analyzed for colony count^35,36^. Briefly, samples were thoroughly mixed and serially ten-fold diluted in sterile water. Aliquots (100 μl) of dilution were cultured on PDA and incubated at room temperature for 24–48 h. The relative spore germination percentage was calculated as relative germination (%) = (the number of germinated spores after plasma or PAW treatment/the number of germinated spores in control (untreated) group) × 100^43^.

### PAW treatment of *A. apis* spore

Aliquots (100 μl) of spore suspension (5 × 10^7^ spores/ml) were added to the 1.5 ml centrifuge tube containing 900 μl of PAW, which resulted in a final concentration of 5 × 10^6^ spores/ml. Spore suspensions in a 900 μl of sterile water were included as control. The samples were mixed and incubated at 30 °C for 1 h. Then, samples were analyzed for spore and colony number as described above.

### Morphological analysis of* A. apis* spore

The morphology of fungal spores was examined by using a scanning electron microscope (Carl Zeiss Auriga, Germany) (The Center for Scientific and Technological Equipment, Suranaree University of Technology, Nakhon Ratchasima, Thailand). Samples were fixed with 2.5% (v/v) glutaraldehyde in phosphate buffer (0.1 M; pH 7.2) for overnight (at 4 °C). The fixed samples were washed three times with phosphate buffer (4 °C; pH 7.2) and then treated with 1% (w/v) osmium tetroxide for 2 h followed by washing with distilled water three times. Subsequently, the samples were dehydrated in a series of increasing acetone concentrations (20%, 40%, 60%, 80%, and 100%) for 15 min each. The samples were dried with critical-point drying (CPD) and covered with a layer of gold 4.5 nm (Leica Sputter Coater EM ACE600, Austria), before undergoing scanning electron microscopy analysis. The scanning electron microscope had an acceleration voltage of 3.0 keV for observations.

### Physicochemical properties of PAW

After PAW generation, pH, conductivity, H_2_O_2_, nitrate and nitrite concentrations were immediately measured. The pH and conductivity of PAW was measured by a pH meter (Mettler Toledo, Switzerland) and EC meter (Apera Instruments, USA), respectively. The amount of H_2_O_2_ was measured using a colorimetric strip test (Supelco MQuant^®^ 1.10337, Merck KGaA, Darmstadt, Germany), whereas the nitrate and nitrite concentrations were measured using nitrate strip test (Supelco MQuant^®^, 1.00020, Merck KGaA, Darmstadt, Germany). The test strip was soaked approximately three seconds into the PAW. Any color change was observed and compared to a color chart on the strip bottle.

### Pathogenicity of plasma-treated spore in honeybee larvae

Fourth or fifth instar larvae were collected from colonies of *A. mellifera* in Phrae, Thailand. Larval age was estimated by size in comparison to 1st–3rd instars. Nine larvae were grafted using a small grafting tool and placed into a 96-well plate provisioned with a worker food mixture (6% d-glucose, 6% d-fructose, 1% yeast extract and 50% royal jelly)^44^ mixed with *A. apis* 1.2 spores. Fungal spores with the concentration of 1.7 × 10^8^ spore/ml were treated with the optimized conditions of gas plasma and PAW from the previous study. Spore suspensions were incubated at 30 °C for 1 h after argon plasma and PAW treatments. Five treatments were assigned as follow: (1) only food mixture (control), (2) food mixture mixed with untreated spores, (3) food mixture mixed with PAW-treated spores, (4) food mixture mixed with direct plasma-treated spores and (5) food mixture mixed with 100 μg/ml of nystatin. A 4-μl of food mixture was daily provided for 7 days. The experiment of each treatment group was run in three replicates (27 larvae/treatment). Mortality of larvae was visually observed and recorded every day. The larvae were considered as dead when they lost their body elasticity or displayed larval color change to brown. Cumulative mortality was calculated per day and expressed as percentage. Afterwards, nine larvae in each treatment were randomly chosen and transferred to PDA plates. PDA plates were incubated at room temperature and mycelial growth was measured daily for 7 days as described above. The percentage of disease inhibition was calculated as percent of inhibition (%) = [(GC-GT)/GC] × 100, where GC (growth control) represents the mean diameter of untreated spores grown in PDA and GT (growth treatment) represents the mean diameter of PAW-treated spores or direct plasma-treated spores or nystatin-treated spores grown in PDA^45,46^.

### Statistics

Statistical analysis was performed using JMP version 11.2 for Mac (SAS Institute Inc., Cary, NC, USA). The normality of the data was checked using the Shapiro–Wilk test. Analysis of variance (ANOVA) followed by Tukey-HSD analysis was applied when data were normally distributed. The non-parametric Kruskal–Wallis test was used to identified if all data had significant differences, followed by a Steel–Dwass posthoc multiple comparisons test to separate means when significance was found. The statistical analyses were regarded as a significant when the p-values was < 0.05.

### Supplementary Information

Supplementary Table S1.

## Data Availability

The datasets used and/or analyzed during the current study are available from the corresponding author on reasonable request.
